# Necrotizing Soft Tissue Infection Post VASER-Assisted Liposuction and Lipofilling: A Case Report

**DOI:** 10.1093/asjof/ojad013

**Published:** 2023-02-06

**Authors:** Mohamed Badie Ahmed, Bara A Shraim, Mutaz Abuelgasim, Atalla Hammouda

## Abstract

Liposuction is a very common aesthetic procedure nowadays. The complication rate is very minimal, and it increases relatively when combined with other procedures. Infection is an expected complication in liposuction and its risk is below 1% in isolated procedures. Although the risk is very low, it might lead to fatal consequences. In this manuscript, the authors present a previously healthy female who presented to the authors’ emergency department post-vibration amplification of sound energy at resonance (VASER)-assisted liposuction and lipofilling done in a private center. Her signs and symptoms started after the procedure and she visited the private center multiple times; however, no significant improvement was felt. Upon her presentation to the authors’ facility, immediate resuscitation was initiated, and she was admitted for further investigations and management. Despite all resuscitation efforts and interventions, the patient’s condition kept deteriorating. She was admitted to the surgical intensive care unit and taken to the operating theater twice with no observed significant improvement. The patient developed septic shock, a multiorgan failure state, followed by cardiac arrest. All resuscitation measures were taken, but the patient could not be revived and was declared dead. Early recognition of signs and symptoms of infection could be lifesaving. Aggressive resuscitation and surgical interventions (extensive debridement and antibiotics) might be necessary to produce successful outcomes.

Level of Evidence: 5

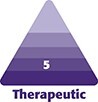

Liposuction is a cosmetic procedure that removes the subcutaneous adipose tissue using a negative pressure suctioning cannula through small incisions made in the skin. The removed fat can be transferred to other areas in the body to enhance body contouring.^1^ Vibration amplification of sound energy at resonance (VASER)-assisted liposuction (VAL) is a newer technique that is being more commonly utilized nowadays. It works by emitting ultrasonic energy that emulsifies and breaks down fat-cell walls/fibrous septae, thus easing fat aspiration.^2^ Complications of liposuction alone are relatively uncommon with a 0.7% risk and reach up to 5% risk when combined with other surgeries. Most common complications include ecchymosis, edema, seroma, infection, and hematoma. The leading cause of death after liposuction is venous thromboembolism with a low risk of 0.03%.^3^ Although the risk of complications is low, many risk factors might play a role in increasing surgical complications such as diabetes, smoking, and cardiovascular diseases. In this case report, we present a young female patient who died post-VAL combined with trochanters lipofilling, and we aim to highlight the deficiencies in the management plan and lessons learned from this case.

## CASE PRESENTATION

A previously healthy 29-year-old female presented to our emergency department complaining of progressive dizziness, shortness of breath, and abdominal pain post-VAL of the abdomen, back, and arms with lipofilling of the trochanters in a private center 12 days before the presentation. Her symptoms were associated with lethargy and palpitations. According to her relatives, postoperatively she received blood transfusion after having her hemoglobin levels drop to 5 g/dL (preoperative: 11 g/dL) and discharged on postoperative day 2. She visited her private center again for concerns of progressive fatigue but was discharged after reassurance. On examination, it was found that the patient was pale and had hypotension with tachycardia and tachypnea. Moreover, there were scattered areas of ecchymosis on the abdomen, back, arms, and thigh, and the surgical site wounds looked infected. The abdomen was tense, warm, and diffusely tender. In addition, there was bilateral lower limb edema. Blood investigations revealed severe anemia (hemoglobin: 6.7 g/dL), high C-reactive protein (330 mg/L), and high lactic acid (5.9 mmol/L). Furthermore, the patient was subjected to abdominal ultrasound, which showed multiple abdominal wall collections, with the largest pocket measuring 8.5 × 7.9 × 2.9 cm with an estimated volume of 104 mL.

**Figure 1.**
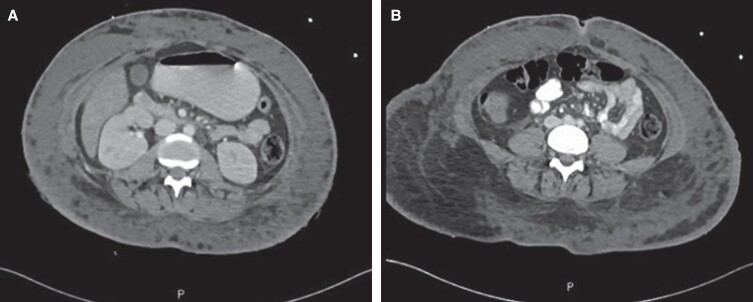
The axial computed tomography scans of a 29-year-old female showing (A) significant fat stranding and (B) edematous changes in the abdominal wall.

After initial improvement with broad spectrum intravenous antibiotics, analgesics, and a blood transfusion, she was shifted to our ward for observation for a provisional diagnosis of infected seroma. A chest X-ray showed bilateral central pulmonary infiltration with left-sided pleural effusion. The abdominal wounds culture revealed pseudomonas aeruginosa. Hence, intravenous amoxicillin-clavulanate (1200 mg) was changed to meropenem 1 g every 8 h and a vancomycin loading dose (1.25 g) followed by 1 g every 8 h. However, after a few hours, the patient became extremely unwell. Upon re-examination, a partial thickness second-degree burn was found over the back and flanks and there were multiple areas of blisters and ecchymosis on the abdomen (∼20% of the total body surface area) from which foul-smelling yellowish discharge was being expressed from ruptured blisters. Subsequently, the patient’s condition started to suddenly deteriorate and she looked drowsy and septic (tachycardia: 120 bpm and hypotension: 98/54 mm Hg), for which rapid response team was activated. Her lactic acidosis worsened (5.9 to 7.2 to 7.9 mmol/L) and the inflammatory markers increased (C-reactive protein: 378 mL/L; procalcitonin: 30 ng/mL). Therefore, a full septic workup was done, and antibiotics were escalated to piperacillin/tazobactam; fluid resuscitation was also initiated. Despite this, there was no clinical improvement, and she was shifted to the surgical intensive care unit (SICU) and intubated for acute respiratory failure. An urgent abdominal computer tomography (CT) scan with intravenous (IV) contrast showed intact intraabdominal organs, diffuse thickening in the abdominal wall, and fat stranding with multifocal areas of fluid collection mainly in the anterior abdominal wall, with the largest pocket measuring ∼7.4 × 2.6 cm (Figure 1).

**Figure 2.**
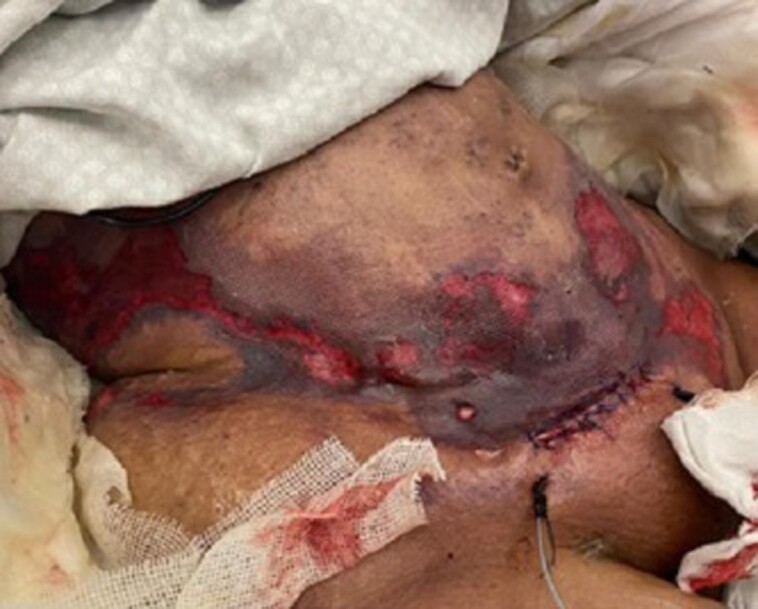
The findings in the abdomen of a 29-year-old female after the first surgical intervention (incision and drainage) showing a second-degree burn with early signs of necrosis in the abdomen and flanks.

Despite this, the patient’s condition kept deteriorating. Her lactate levels worsened to 11 mmol/L, procalcitonin levels reached >100 ng/mL, and she developed severe hypoglycemia and oliguria. A re-examination showed that the abdomen was becoming more tense with necrotic tissue and blisters and the intraabdominal pressure was elevated. At this stage, the patient was urgently shifted to the operating theater (OT) for incision and drainage of body collections. A transverse incision along the lower abdomen and the right trochanter (lipofilling) was done; 2500 mL of foul-smelling pus mixed with serosanguineous fluid was evacuated (Figure 2). Culture of the pus and wounds showed *Escherichia coli*. The general condition of the patient did not improve and kept deteriorating. Based on SICU assessment, the patient had developed multiorgan failure likely from abdomen necrotizing fasciitis (NF). Thus, she was shifted to OT after 12 h from the previous operation due to septic shock and a suspicion of NF. Extensive debridement of the necrotic skin and subcutaneous tissue in the liposuction and lipofilling areas along with fasciotomy of the abdominal fascia were done. The abdominal fascia was intact with no signs of necrosis. Postoperatively, there was still no improvement in the patient's condition, and she developed disseminated intravascular coagulation. A few hours later, she suffered cardiac arrest and could not be revived.

### Ethical Approval

As per our hospital policy, no consent is required as long as patient data are deidentified and the report is approved by the Hamad Medical Corporation—Medical Research Center (HMC-MRC). This manuscript is approved by the HMC-MRC with reference number: MRC-04-22-846.

## DISCUSSION

Ultrasound-assisted lipectomy or the newly named VAL is a technique in liposuction that is employed to emulsify fat tissue using ultrasound energy through a metal rod before aspiration. Multiple studies compared this technique to suction-assisted lipectomy to assess their risks and benefits. A few studies concluded that VAL was superior in terms of reduction in blood loss.^4–6^ On the other hand, multiple studies reported an equivocal outcome between both techniques in terms of complications and results.^7–9^

Necrotizing soft tissue infection (NSTI) is a severe life-threatening infection characterized by widespread tissue destruction, systemic toxicity, hemodynamic instability, multiorgan failure, and high mortality.^10^ NF, a type of NSTI, spreads to the underlying tissue and is characterized by a friable superficial fascia and grayish inflammatory fluid with the absence of pus upon surgical exploration.^10^ In this paper, we report a patient who died post-VAL of the abdomen, back, and arms with lipofilling of the trochanters due to severe infection that led to septic shock followed by multiorgan failure and cardiac arrest. The patient sought medical attention initially from the private center, but according to her, she was dismissed, and no proper investigations were done. Her symptoms kept worsening until she presented to our emergency in a critical condition. Her clinical picture was initially pointing toward NF until she was operated, and intraoperatively, the abdominal fascia was intact, which suggested only an NSTI.

Infection is an expected complication post-liposuction procedure with a risk of 0.1% in isolated procedures, and it increases to 0.7% in multiple procedures.^3^ A more severe form of infection might lead to NF, which is a severe condition that includes a rapid spread of the infection from the subcutaneous tissue to the underlying superficial and deep fascial planes. The mortality rate in such cases might reach up to 20% to 40%.^11^ A review paper published by Marchesi et al in 2017 reported 15 cases of NF postaesthetic surgery, with liposuction being the most affected procedure in >50% of the cases. All patients presented with signs and symptoms in the first 3 to 4 days postoperatively, except 2 patients, who had delayed presentation for more than 30 days.^12^ In our patient, the signs and symptoms started within the first 2-3 days postoperatively, and these were fever, local edema, erythema, tenderness, and pain. The patient’s condition kept deteriorating until she developed sepsis and blisters and bullae over her abdomen, which initially implied NF; thus, she was shifted to the theater for debridement. However, intraoperatively, the abdominal fascia was intact, which did not support a diagnosis of NF. Nevertheless, the blisters and bullae were most likely due to excessive use of VASER in her primary procedure. Despite surgical intervention, the patient died 9 h after the second surgical intervention.

This devastating consequence could have been prevented if there was a high index of suspicion of NSTI and NF and early intervention to avoid their ominous consequences. In this context, the modified laboratory risk indicator for NF (LRINEC) may be a valuable tool for consideration in rapid recognition of NF and subsequent initiation of surgical intervention to improve disease outcomes.^13^ Moreover, the delayed presentation of the case to the emergency department limited our management options and left us with interventions that were less likely to succeed. The mainstay of treatment of NSTI includes early antibiotic administration and urgent extensive debridement. Moreover, a case report demonstrated that negative pressure wound therapy was of high importance in the management of NSTI.^14^ In comparison with other cases, we find our case unique, because, to the best of our knowledge, it is the first case to report death as a complication of VAL and lipofilling, because using VASER in this case gave a clinical picture that mimicked NF mainly due to the formation of blisters and a mixed partial thickness burn seen on the abdominal wall. We suspect that she underwent massive liposuction (specific numbers could not be obtained), which precipitated her cascade of complications. The take-home message from this case is that surgeons should bear in mind that early signs and symptoms of fulminant infection or NF are usually nonspecific. Thus, as soon as there is a suspicion, immediate intravenous antibiotics and surgical intervention should be initiated without delay.

## CONCLUSIONS

In conclusion, liposuction is one of the most common cosmetic procedures performed worldwide and it can be associated with severe complications. Moreover, surgeons should have a high index of suspension to detect abnormal signs and symptoms during early postoperative days and be mindful about the technique and volume of liposuction planned for patients. Rapid recognition of postoperative infection or NF and early intervention are key factors to prevent devastating outcomes.
